# Risk factors for early local lymph node recurrence of thoracic ESCC after McKeown esophagectomy

**DOI:** 10.3389/fsurg.2022.1043755

**Published:** 2023-01-06

**Authors:** Liang Dai, Yong-Bo Yang, Ya-Ya Wu, Hao Fu, Wan-Pu Yan, Yao Lin, Zi-Ming Wang, Ke-Neng Chen

**Affiliations:** Key Laboratory of Carcinogenesis and Translational Research (Ministry of Education), The First Department of Thoracic Surgery, Peking University Cancer Hospital and Institute, Peking University School of Oncology, Beijing, China

**Keywords:** early local lymph node recurrence, mckeown esophagectomy, risk factors, esophageal squamous cell carcinoma, prognosis of esophageal cancer

## Abstract

**Objectives:**

Even underwent radical resection, some patients of thoracic esophageal squamous cell carcinoma (ESCC) are still exposed to local recurrence in a short time. To this end, the present study sought to differentiate patient subgroups by assessing risk factors for postoperative early (within one year) local lymph node recurrence (PELLNR).

**Methods:**

ESCC patients were selected from a prospective database, and divided into high- and low-risk groups according to the time of their local lymphatic recurrence (within one year or later). Survival analysis was conducted by the Cox regression model to evaluate the overall survival (OS) between the two groups. The hazard ratio (HR) and 95% confidence interval (CI) of different variables were also calculated. Logistic regression analysis was used to explore the high-risk factors for PELLNR with the odds ratio (OR) and 95% CI calculated.

**Results:**

A total of 432 cases were included. The survival of patients in the high-risk group (*n* = 47) was significantly inferior to the low-risk group (*n* = 385) (HR = 11.331, 95% CI: 6.870–16.688, *P* < 0.001). The 1-year, 3-year, and 5-year OS rate of the patients in high/low-risk groups were 74.5% vs. 100%, 17% vs. 88.8%, and 11.3% vs. 79.2%, respectively (*P* < 0.001). Risk factors for local lymph node recurrence within one year included upper thoracic location (OR = 4.071, 95% CI: 1.499–11.055, *P* = 0.006), advanced T staging (pT3–4, OR = 3.258, 95% CI: 1.547–6.861, *P* = 0.002), advanced N staging (pN2–3, OR = 5.195, 95% CI: 2.269–11.894, *P* < 0.001), and neoadjuvant treatment (OR = 3.609, 95% CI: 1.716–7.589, *P* = 0.001). In neoadjuvant therapy subgroup, high-risk group still had unfavorable survival (Log-rank *P* < 0.001). Multivariate analysis demonstrated that upper thoracic location (OR = 5.064, 95% CI: 1.485–17.261, *P* = 0.010) and advanced N staging (pN2–3) (OR = 5.999, 95% CI: 1.986–18.115, *P* = 0.001) were independent risk factors for early local lymphatic recurrence. However, the cT downstaging (OR = 0.862, 95% CI: 0.241–3.086, *P* = 0.819) and cN downstaging (OR = 0.937, 95% CI: 0.372–2.360, *P* = 0.890) for patients in the neoadjuvant subgroup failed to lower PELLNR. The predominant recurrence field type was single-field.

**Conclusions:**

Thoracic ESCC patients with lymph node recurrence within one year delivered poor outcomes, with advanced stages (pT3–4/pN2–3) and upper thoracic location considered risk factors for early recurrence.

## Introduction

Esophageal squamous cell carcinoma (ESCC) is the most common esophageal malignancy, featuring an Asian lineage and a thoracic location as the most common circumstances (1, 2). Multidisciplinary treatment is the generally accepted treatment strategy for locally advanced ESCC, with surgery considered a key component of a comprehensive treatment framework (3, 4). However, even after radical resection, some patients still face local recurrence in a short period (5, 6). Particularly, early local lymphatic recurrence within one year after surgery is the main reason for postoperative failure and poor prognosis for long-term survival (7). In addition, early recurrence raises questions among doctors and patients about the role of surgery in comprehensive treatment of ESCC. However, the clinical factors affecting early postoperative local lymphatic recurrence are inconclusive, thereby resulting in the lack of support for adjuvant treatment of patients undergoing R0 resection in clinical guidelines, including *The National Comprehensive Cancer Network* (8). It is hereby hypothesized that distinct clinicopathological characteristics determine the likelihood of early local lymphatic recurrence within one year after radical resection. Two subgroups of patients are speculated to require different diagnosis and treatment programs. Thus, a retrospective review was hereby conducted upon the prospective database of the Thoracic Surgery Department I of Peking University Cancer Hospital, taking thoracic ESCC patients having undergone radical esophagectomy as subjects. Clinicopathological factors and follow-up information were reviewed to assess risk factors for early local lymphatic recurrence, and long-term prognostic characteristics were examined to clarify the early-recurrence subgroup of patients.

## Methods

### Characteristics of the database

Eligible patients were screened from the prospective ESCC database of our department. In accordance with the Institutional Review Board, the informed consent requirement was waived for this study. The database was established in 2000, and is provided with the following characteristics:

1) It featured a high-level standardization. Data collection was designed as a pull-down menu of standardized items, which avoided varying physician descriptions.
2) Baseline data must be entered before initial treatment, and the pre-operative data including the re-staging information must be entered before surgery. The intraoperative findings (operation notes) must be completed before the patient leaves the operating room, and discharge notes must be entered before the patient leaves the hospital. Outpatient follow-up information must be entered in real-time.
3) The pre-treatment/pre-operation examinations included gastroscopy with tumor biopsy and pathological diagnosis. The staging and quantitative examinations included gastroscopy bronchoscopy (middle or upper thoracic ESCC), chest/abdominal contrast CT scan, abdominal ultrasound, and cervical-supraclavicular ultrasound, and upper gastrointestinal barium meal. Since its establishment in 2012, whole-body PET/CT and ultrasound endoscopy have been performed routinely.
4) Patients were staged according to *The 7^th^ Edition of the Union for International Cancer Control (UICC)/American Joint Committee on Cancer (AJCC) TNM* staging system for esophageal cancer (9).
5) Locally advanced patients (cT3∼4a or cN+) received neoadjuvant treatment, predominantly induction chemotherapy; the regimens were dual drug combinations based on platinum, 95% of which were paclitaxel combined with cisplatin. All patients underwent surgery 4∼6 weeks after neoadjuvant chemotherapy.
6) Follow-up was defined as outpatient visit with standard examinations. Follow-up evaluation consisted of interviews at 3-month intervals for 2 years, then at 6-month intervals for 3 years, and ﬁnally at 12-month intervals until death. Outpatient follow-up visits included records of symptoms and ﬁndings of physical examinations. Objective examinations included chest CT scan, barium upper esophagography, abdominal and cervical ultrasound, and gastroscopy, if necessary. Since 2010, some subjects have undergone positron emission tomography-computed tomography (PET/CT) examinations.
7) Local lymph node recurrence was defined as regional lymph nodes within the surgical field, while lymph node-recurrent regions were classified as cervical-supraclavicular lymph node, mediastinal lymph node, and abdominal lymph node according to locations of the lymph nodes. The standard for recurrence was newly found enlarged lymph nodes (minimal diameter > 10 mm) on the follow-up cervical-supraclavicular region by physical examination/ultrasound/CT, chest by CT, and abdominal by CT/ultrasound, which was hypermetabolic on PET/CT.

### Inclusion and exclusion criteria

Inclusion criteria: 1) Patients having received surgery between January 1 2010 and April 30 2017; 2) Treatment naïve patients before visiting us; 3) Pathologically confirmed squamous cell carcinoma; and 4) Patients having undergone the McKeown (open/minimal invasive) procedure and R0 resection (en-bloc) with at least two-field lymph node dissection.

Exclusion criteria:1) Patients with cervical esophageal cancer; 2) Patients with distant metastases or local recurrence plus distant metastases as the first recurrence; 3) Patients exposed to anastomotic recurrence; 4) Patients subject to perioperative death (died within 90 days after surgery); 5) Patients having died from reasons other than cancer; 6) Patients having received adjuvant radiotherapy after surgery; or 7) Patients presenting other malignancies at the time of ESCC.

Herein, a total of 432 cases were ultimately surveyed. Based on the observation of the lymph node recurrence risk of esophageal cancer in this center, it was found that one year after surgery was the highest risk of recurrence, which was thus divided into a high-risk group (local lymphatic recurrence within one year) and a low-risk group (local lymphatic recurrence after one year) (Supplementary Appendix S1).

### Statistics

SPSS 25.0 software (IBM Corp, Armonk, NY) was used for statistical analysis; the chi-square test or Fisher's exact probability method was used for numerical data comparisons, and the rank sum test was used for ranked data comparison. The correlation between different parameters was analyzed using Pearson correlation analysis, and the Kaplan-Meier curve was used to analyze the survival of patients. Intergroup survival analysis was completed using the Log-Rank method. Multivariate survival analysis was conducted based on the Cox regression model. The hazard ratio (HR) and 95% confidence interval (CI) of different variables were also calculated. A logistic regression model was used to evaluate risk factors for recurrence within one year with the odds ratio (OR) and 95% CI calculated. The *P* value less than 0.05 was defined as statistical significance.

## Results

### General characteristics of the patients

A total of 432 cases were selected for this study, of which, 327 (78.2%) were male and 105 (21.8%) were female, with a median age of 60 (range: 39–80). Besides, 216 patients (50%) received neoadjuvant therapy, and 17 (8%) obtained pCR as confirmed by postoperative pathological examination. The numbers of cases with Stage I, II, and III were 122 (28.2%), 186 (43.1%), and 107 (24.8%), respectively. The follow-up rate was 91.2%, with 38 cases lost to follow-up. The median follow-up time was 41.9 months (3.2 months to 116.5 months). At the last follow-up, 114 cases (26.4%) had local recurrence, and 93 (49.2%) died. Upon recurrence, 76 cases (66.7%) received chemo/chemoradiotherapy, and 38 (33.3%) received supportive treatment only. The general clinicopathological data of the high-risk group vs. the low-risk group and the neoadjuvant treatment group vs. the direct surgery group are shown in Supplementary Appendices S2, S3.

### Survival analysis

**Figure 1:**
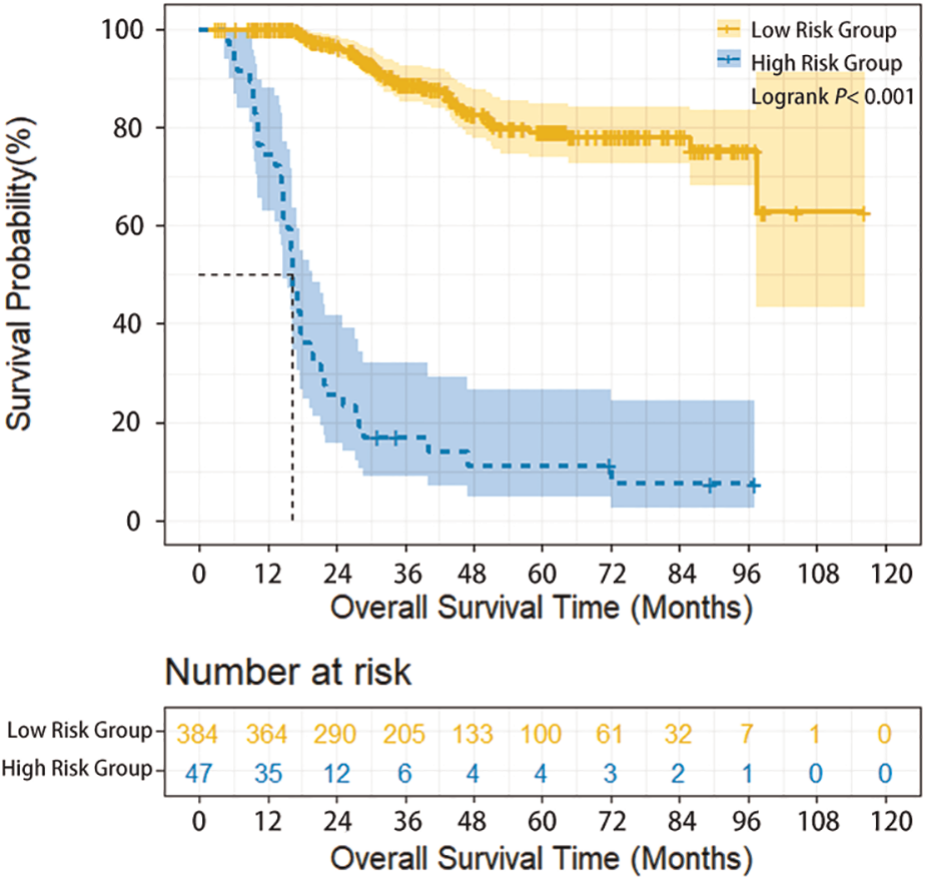
Overall survival of ESCC patients with different lymphatic recurrence risk in whole group. Overall survival of high-risk patients was significantly worse compared with low-risk patients in the whole group (*P* < 0.001).

The survival of high-risk patients (47 cases, 10.9%) was significantly worse than that of low-risk patients (385 cases, 90.1%) (HR = 11.331, 95% CI: 6.870–16.688, *P* < 0.001) (Table 1). The 1-year, 3-year, and 5-year overall survival (OS) of high-risk and low-risk patients was 74.5% vs. 100%,17% vs. 88.8%, and 11.3% vs. 79.2% (*P* < 0.001), respectively (Figure 1).

**Table 1:** Multivariate COX regression overall survival analysis.

Item	Multivariate		
	OR	95% CI	*P*
Sex (Male vs. Female)	1.177	0.700–1.981	0.539
Age (>60 year vs. ≤ 60 year)	0.844	0.549–1.298	0.441
Location			0.771
L1 vs. L3	1.064	0.607–1.867	0.828
L2 vs. L3	0.879	0.459–1.683	0.697
Neoadjuvant therapy (Yes vs. No)	1.189	0.732–1.931	0.485
Lvi (Yes vs. No)	0.880	0.496–1.561	0.662
pT (T3-4 vs. T1-2)	2.493	1.562–3.980	0.000
pN (N2-3 vs. N0-1)	2.223	1.241–3.982	0.007
Lymph nodes dissected (>20 vs. ≤ 20)	1.392	0.905–2.141	0.132
High-risk vs. low-risk group	11.331	6.870–18.688	0.000

OR, odds ratio; CI, confidence interval; L1, upper thoracic location; L2, middle thoracic location; L3, lower thoracic location; Lvi, lymph-vascular invasion.

### Analysis for risk factors of PELLNR within one year

Upper thoracic location (OR = 4.071, 95% CI: 1.499–11.055, *P* = 0.006), advanced T staging (pT3–4) (OR = 3.258, 95% CI: 1.547–6.861, *P* = 0.002), advanced N staging (pN2–3) (OR = 5.195, 95% CI: 2.269–11.894, *P* < 0.001), and neoadjuvant therapy (OR = 3.609, 95% CI: 1.716–7.589, *P* = 0.001) were found independent risk factors for early local lymphatic recurrence *via* multivariate analysis (Table 2).

**Table 2:** Results of univariate and multivariate analyses of risk factors for PELLNR in ESCC patients with radical esophagectomy.

Item	Univariate			Multivariate		
	HR	95% CI	*P*	HR	95% CI	*P*
Sex (Female vs. Male)	1.218	0.616–2.405	0.571			
Age (>60 year vs. ≤ 60 year)	0.624	0.338–1150	0.131			
Smoker (Yes vs. No)	1.107	0.585–2.096	0.755			
Location (L1 vs. L2+L3)	3.32	1.319–8.359	0.11	4.071	1.499–11.055	0.006
Multiple primary tumor (Yes vs. No)	1.644	0.599–4.509	0.334			
Neoadjuvant therapy (Yes vs. No)	3.287	1.656–6.525	0.001	3.609	1.716–7.589	0.001
Approach (VATS vs. Open)	0.741	0.378–1.453	0.383			
Lymph nodes dissected (>20 vs. ≤ 20)	1.125	0.613–2.064	0.704			
Lvi (Yes vs. No)	2.168	1.097–4.284	0.026	1.262	0.557–2.857	0.577
pT (T3-4 vs. T1-2)	4.423	2.226–8.788	<0.001	3.258	1.547–6.861	0.002
pN (N2-3 vs. N0-1)	7.127	3.459–14.682	<0.001	5.195	2.269–11.894	<0.001
Serious complication (Yes vs. No)	1.518	0.556–4.142	0.415			
Postoperative adjuvant therapy (Yes vs. No)	1.444	0.783–2.663	0.239			

HR, hazard ratio; CI, confidence interval; L1, upper thoracic location; L2, middle thoracic location; L3, lower thoracic location; VATS, video-assisted thoracoscopic surgery; Lvi, lymph-vascular invasion.

### Subgroup analysis for patients with neoadjuvant therapy

**Figure 2:**
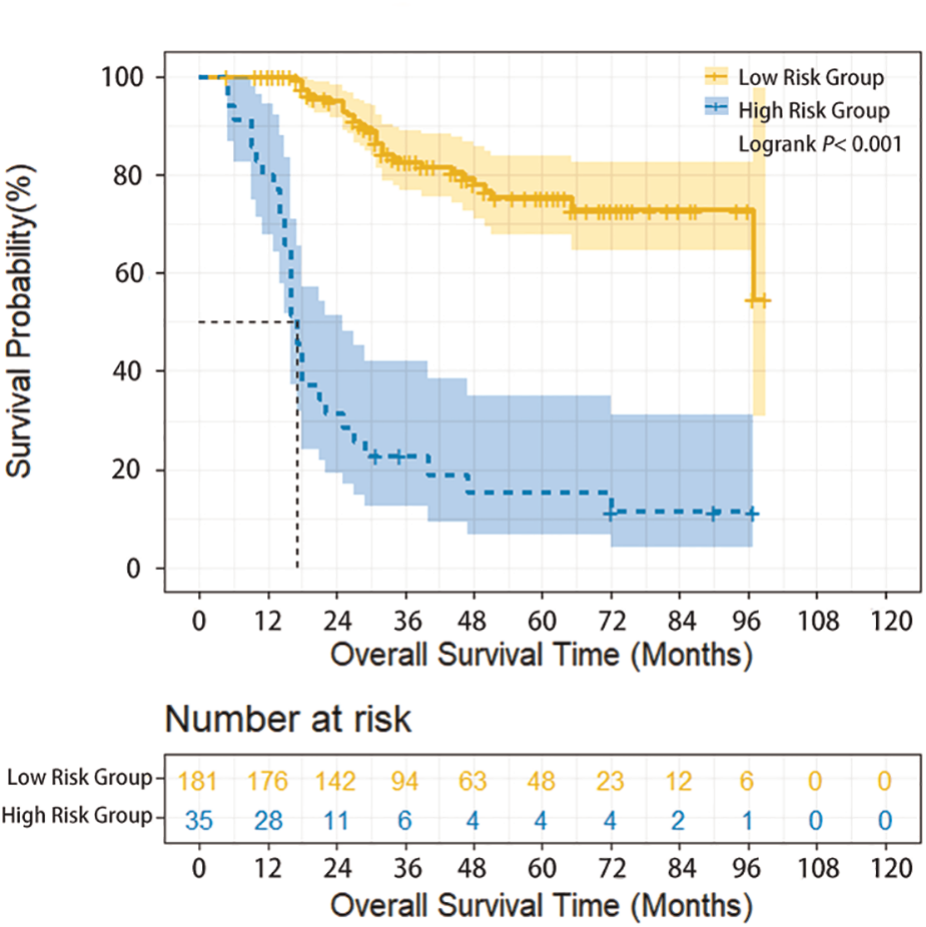
Overall survival of ESCC patients with different lymphatic recurrence risk in neoadjuvant treatment subgroup. Overall survival of high-risk patients was significantly worse compared with low-risk patients in the neoadjuvant treatment subgroup (*P* < 0.001).

Herein, 206 (95.37%) of 216 patients with neoadjuvant chemotherapy were treated with TP regimen (paclitaxel/nab-paclitaxel+cisplatin) and 22 (10.19%), 169 (78.24%), 17 (7.87%) and 8 (3.70%) patients underwent 1, 2, 3 and 4 cycles of preoperative treatment, respectively. Compared with patients having undergone directly surgery, patients who received neoadjuvant therapy had more advanced stages, and the proportion of cN+cases in the two subgroups was 16.2% and 64.8%, respectively. In addition, for neoadjuvant therapy cases, high-risk group (35 cases, 16.2%) had poorer survival compared with low-risk group (181 cases, 83.8%) (HR = 7.991, 95% CI: 4.482–14.248, *P* < 0.001). The 1-year, 3-year, and 5-year OS of high-risk and low-risk patients were 80% vs. 100%, 15.2% vs. 82.6%, and 10.2% vs. 75.2%, respectively (*P* < 0.001) (Figure 2). Multivariate analysis demonstrated that upper thoracic location (OR = 5.064, 95% CI: 1.485–17.261, *P* = 0.010) and advanced N staging (pN2–3) (OR = 5.999, 95% CI: 1.986–18.115, *P* = 0.001) were independent risk factors for early local lymphatic recurrence. However, after neoadjuvant therapy, cT downstaging (OR = 0.862, 95% CI: 0.241–3.086, *P* = 0.819) or cN downstaging (OR = 0.937, 95% CI: 0.372–2.360, *P* = 0.890) failed to lower the risk for early lymphatic recurrence (Table 3).

**Table 3:** Results of univariate and multivariate analyses of risk factors for PELLNR in ESCC patients with neoadjuvant therapy.

Item	Univariate			Multivariate		
	HR	95% CI	*P*	HR	95% CI	*P*
Sex (Female vs. Male)	0.881	0.358–2.167	0.783			
Age (>60 year vs. ≤ 60 year)	0.547	0.260–1.152	0.112			
Smoker (Yes vs. No)	1.127	0.751–1.692	0.563			
Location (L1 vs. L2+L3)	4.435	1.424–13.812	0.01	5.064	1.485–12.261	0.01
Multiple primary tumor (Yes vs. No)	1.118	0.304–4.116	0.866			
Approach (VATS vs. Open)	0.827	0.373–1.834	0.64			
Lymph nodes dissected (>20 vs. ≤ 20)	1.017	0.492–2.103	0.964			
Lvi (Yes vs. No)	3.312	1.428–7.684	0.005	2.117	0.777–5.768	0.142
pT (T3-4 vs. T1-2)	3.301	1.498–7.275	0.003	2.73	0.760–9.807	0.124
pN (N2-3 vs. N0-1)	8.922	3.466–22.963	<0.001	5.999	1.986–18.115	0.001
cT down staging	0.413	0.188–0.910	0.028	0.862	0.241–3.086	0.819
cN down staging	0.516	0.234–1.138	0.101	0.937	0.372–2.360	0.89
Serious complication (Yes vs. No)	1.212	0.327–4.495	0.774			
Postoperative adjuvant therapy (Yes vs. No)	1.1	0.514–2.354	0.806			

HR, hazard ratio; CI, confidence interval; L1, upper thoracic location; L2, middle thoracic location; L3, lower thoracic location; VATS, video-assisted thoracoscopic surgery; Lvi, lymph-vascular invasion.

### Lymph node dissection site and common sites for local lymphatic recurrence

All the patients in the study had two- or three-field lymph node dissection. Patients who had mediastinal lymph node dissection mainly included 304 cases (70.37%) with left and right recurrent laryngeal nerve lymph nodes, 419 cases (96.99%) with subcarinal lymph nodes, 432 cases (100%) with paraesophageal lymph nodes, and 323 cases (74.77%) of superior phrenic lymph nodes; patients who had abdominal lymph node dissection mainly included 422 (97.69%) with right cardiac lymph nodes, 422 patients (97.69%) with left cardiac lymph nodes, 422 patients (97.69%) with gastric lesser curvature lymph nodes, 422 patients (97.69%) with left gastric periarterial lymph nodes; 19 patients (4.39%) underwent cervical lymph node dissection. The most common sites for local lymphatic recurrence were mediastinal lymph nodes (74 cases, 17.1%), cervical lymph nodes (44 cases, 10.2%), and abdominal lymph nodes (19 cases, 4.4%), successively. The predominant field type for recurrence was single-field, with 92 (21.3%) cases found to have a single-field recurrence, and 22 (5.1%), multiple-field recurrence.

### Comment

A high-quality prospective database is important for a reliable retrospective study, and standardized terms, prospective maintenance, and formatted content are the sole requirements for data quality. Herein, the original data (including image series) were traceable for each patient in our study. In order to avoid the interference of different lymph node dissection ranges of different procedures, the inclusion/exclusion criteria were hereby designed to avoid possible ambiguous factors to affect the survival. For example, only patients having undergone the McKeown (open/minimally invasive) procedure and en-bloc resection were included (10). All patients had at least two-field (chest and abdomen) lymph node dissection and R0 resection (11). Patients with cervical esophageal cancer and those who either had simultaneous distant metastases as the first recurrence, anastomotic recurrence or underwent postoperative supplementary radiation, were excluded (12, 13). Even though, 10.9% of PELLNR cases were still observed. Although 87% of the patients received radiotherapy/chemotherapy upon the detection of recurrence, the long-term prognosis was still far worse than that of the low-risk group. In this case, it was thought that the long-term survival of ESCC patients could be improved by strengthening local control measures to control regional lymph node recurrence better.

Upper thoracic location, advanced T/N staging, and preoperative therapy were found independent risk factors for early local recurrence *via* multivariate analysis, and such a finding is provided with the following clinical implications:

1. Cervical lymph node dissection should be emphasized for upper thoracic ESCC. Japanese surgeons believe that cervical lymph node dissection should be routinely performed to reduce the local recurrence rate of ESCC in the upper thorax (14, 15). However, in this study, cervical lymph node dissection was only performed for those with clinical suspicious lymph node metastases (only 19 cases), which might be one of the reasons for the higher risk of ESCC in the upper thorax.
2. Staging of esophageal cancer is hindered by the low coincidence rate between clinical and postoperative pathological staging, and methods from multiple perspectives are thus required for more accurate staging. Compared with other solid malignancies such as lung cancer, various preoperative staging methods for esophageal cancer are subject to certain limitations, thereby affecting the accuracy of clinical staging, also the differentiation of the postoperative curative effect (16, 17). For this reason, the pathological staging was still hereby used to reflect the malignant degree of the tumor. According to multivariate analysis, patients with more advanced stages (pT3–4/pN2–3) presented higher infiltration and metastatic ability of the tumor and were more likely to have PELLNR.
3. The importance of re-staging after induction therapy needs to be emphasized. Neoadjuvant chemotherapy could reduce the tumor size, eliminate potential metastases, and downstage the tumor, thus reducing postoperative recurrence and metastasis, and improving the long-term survival of the patients (3). In order to minimize the impact of selection bias on the results, subgroup analysis was performed for patients with neoadjuvant therapy. The results showed that upper thoracic location and pN2–3 were still risk factors for PELLNR. However, the responses to neoadjuvant therapy (cT downstaging and cN downstaging) were not independent risk factors for PELLNR. The potential reasons were: first, the small sample size limited the influence of different tumor responses on the risk of PELLNR; second, the accuracy of clinical evaluation for the efficacy of neoadjuvant therapy was still unsatisfactory.
4. Efforts should be made to introspect the survival benefits of chemotherapy alone and provide more evidence for the effect of induction chemotherapy alone and induction chemoradiotherapy. In the current study, those having received neoadjuvant therapy were more likely to have PELLNR. Although further analysis showed that the proportion of cN+was higher in the neoadjuvant therapy group (64.8%) compared with that in the upfront surgery group (16.2%), the benefit of neoadjuvant therapy could not counteract the influence of the advanced stage. Additional research should focus on the differences in the clinical benefit of curative chemoradiotherapy or surgery for the patients who failed to get downstaging after induction chemotherapy, and finally provide a reference for clinicians to establish the corresponding treatment strategies for different patient subgroups (8, 9).

## Limitations of the study

First, the retrospective nature of the study determined the inevitable selection bias. For example, most patients who had received neoadjuvant therapy due to an advanced disease still had an early recurrence. Second, although the type of esophagectomy was limited to the McKeown procedure, and the resection pattern and dissection range of lymph nodes were strictly controlled, the influence of surgical quality on the recurrence could not be assessed. Third, the sample size was rather limited, and the single-center nature of the hereby selected data might have biased the interpretation of the results.

In summary, the results showed that patients with PELLNR had poorer survival and that upper thoracic location and advanced T/N staging (pT3–4/pN2–3) were the risk factors for PELLNR. For patients having received induction therapy due to advanced disease at the baseline, re-staging after neoadjuvant treatment should be reinforced to distinguish those who could oncologically benefit from surgery and those with only technically resectable tumors.

## Data Availability

The original contributions presented in the study are included in the article/**Supplementary Material**, further inquiries can be directed to the corresponding author/s.
