# Supplementary figures and images for: Risk factors for early local lymph node recurrence of thoracic ESCC after McKeown esophagectomy
Source: Front Surg. 2023 Jan 6;9:1043755. doi: 10.3389/fsurg.2022.1043755 (PMC9852523; doi:10.3389/fsurg.2022.1043755)

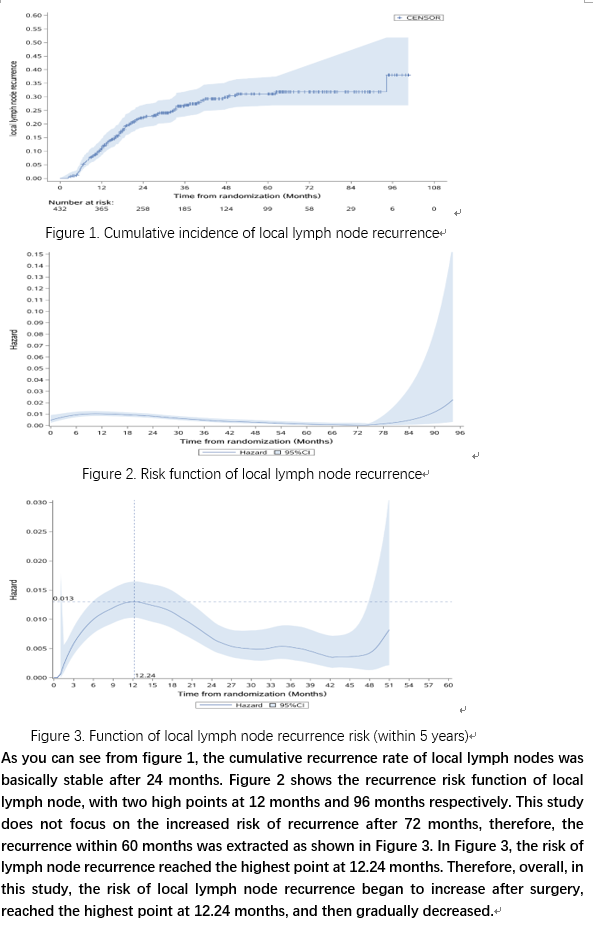

Supplement: Supplementary file 1 [file Image1.png]
